# Role of Teneurin C-Terminal Associated Peptides (TCAP) on Intercellular Adhesion and Communication

**DOI:** 10.3389/fnins.2022.868541

**Published:** 2022-05-02

**Authors:** Thomas L. Dodsworth, David A. Lovejoy

**Affiliations:** Department of Cell and Systems Biology, University of Toronto, Toronto, ON, Canada

**Keywords:** peptides, GPCR, energy, latrophilin, evolution, calcium, cytoskeleton, brain

## Abstract

The teneurin C-terminal associated peptides (TCAP) are encoded by the terminal exon of all metazoan teneurin genes. Evidence supports the liberation of a soluble TCAP peptide either by proteolytic cleavage from the mature transmembrane teneurin protein or by a separately transcribed mRNA. Synthetic versions of TCAP, based on its genomic structure, are efficacious at regulating intercellular communication by promoting neurite outgrowth and increasing dendritic spine density *in vitro* and *in vivo* in rodent models. This is achieved through cytoskeletal re-arrangement and metabolic upregulation. The putative receptors for TCAPs are the latrophilin (LPHN) family of adhesion G-protein coupled receptors, which facilitate TCAP’s actions through G-proteins associated with cAMP and calcium-regulating signalling pathways. The teneurin/TCAP and latrophilin genes are phylogenetically ancient, likely serving primitive functions in cell adhesion and energy regulation which have been since adapted for a more complex role in synaptogenesis in vertebrate nervous systems.

## Introduction to the Teneurin C-Terminal Associated Peptides (TCAPs)

The teneurin C-terminal associated peptides (TCAPs) were initially discovered in a screen of a rainbow trout hypothalamic cDNA library for genes related to the corticotropin releasing factor (CRF) family. In this study, Qian et al. (2004) used a hamster urocortin cDNA probe to identify a neuropeptide-like region representing the C-terminal 40 amino acids of rainbow trout teneurin-3, named in accordance with its placement in this gene. Vertebrates have four paralogous forms of this 40–41 amino acid peptide, with each paralogue found at the C-terminus of one of the teneurin genes, hence their nomenclature as TCAPs 1–4. The teneurins were initially identified in a screen for proteins related to the tenascins and were named based on their high expression in the central nervous system (CNS; Baumgartner et al., 1994; review: Baumgartner and Wides, 2019). Teneurins possess a functional importance in development and maintenance of the nervous system. Homophilic and heterophilic teneurin interactions mediate process outgrowth, cell adhesion and synaptic organization in both vertebrates and invertebrates (reviews: Mosca, 2015; Tucker, 2018). Some paralogues have specific functions: for example, teneurin-3 is important for neuronal wiring in the developing visual system and hippocampus (Young and Leamey, 2009; Berns et al., 2018). The teneurins possess many functional domains within their extracellular C-terminal region which will not be discussed in detail here, as we will primarily focus on the function on the TCAP region and its ability to modulate neuronal connectivity.

The TCAP sequence is located within the terminal exon of the teneurin mRNA and is flanked by a prohormone convertase (PC)-like cleavage signal at the 5′ end and a glycine-lysine-arginine (GKR) amidation motif at the 3′ end which precedes the stop codon and subsequent 3′ untranslated region (Qian et al., 2004; Wang et al., 2005). Additional basic/dibasic cleavage sites are located further upstream of TCAP, but only some are conserved (Chand et al., 2013). These conserved cleavage sites indicate that TCAPs may be cleaved from teneurin and act on receptors in an autocrine or paracrine manner (Chand et al., 2013). Based on the criteria outlined by Seidah and Chrétien (1997), the translated portion of the terminal exon of mouse teneurin-1 possesses five potential sites for cleavage by prohormone convertases, where three of these sites are conserved across all teneurins (Chand et al., 2013). TCAP is exteriorly oriented when teneurin is folded, and therefore the exposed helix at the N-terminus of TCAP could be accessed by proteases (Jackson et al., 2018; Li et al., 2018). There is also evidence that some TCAP paralogues may be functionally processed as an individual peptide through separate transcription and translation (Chand et al., 2013). *In situ* hybridization experiments have revealed some differences between the expression of TCAP-1 and teneurin-1 in the rodent brain; specifically, TCAP-1 has high expression in the diencephalon and limbic system, whereas teneurin-1 does not (Zhou et al., 2003; Wang et al., 2005). Similarly, immunocytochemical labelling has shown distinct cellular localization of teneurin-1 and TCAP-1 in mouse hippocampal E14 cells, where both co-localize on the plasma membrane, but TCAP-1 labelling alone is detected diffusely in the cytosol (Chand et al., 2013). Northern blot studies suggest that the terminal exons of teneurin-1 and 3, which contain TCAP-1 and 3, can be transcribed independently from their full-length teneurin genes (Chand et al., 2013). Using 5′ rapid amplification of cDNA ends polymerase chain reaction (5′RACE PCR), a 485-base pair transcript that contains TCAP-1 was isolated from whole mouse brain RNA. The separate TCAP-1 mRNA produces a 13-kDa propeptide when hypothetically translated from the first ATG signal. In fact, Chand et al. (2013) did identify a 13-kDa TCAP-1-immunoreactive band by Western blot, but it is unclear if this band is the product of TCAP-1 mRNA translation or the product of teneurin cleavage at one of the prohormone convertase motifs. Regardless, the putative translation product of the TCAP-1 mRNA does not code for a signal peptide, which could explain the diffuse immunoreactivity of TCAP-1 in E14 cells as the propeptide would be translated by free ribosomes and remain in the cytosol. This contrasts with the teneurin-derived form, which enters the secretory pathway and becomes associated with the extracellular face of the plasma membrane due to the type II orientation of the teneurin protein. Given the distinction in cellular localizations, a separately transcribed form of TCAP-1 may have physiological functions that are distinct from the teneurin-1-derived form.

**FIGURE 1:**
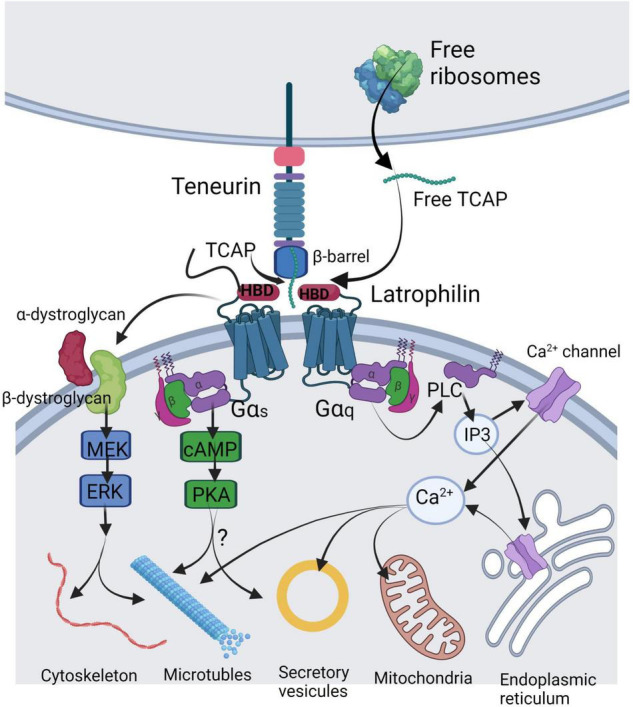
Illustration of the mechanisms of TCAP release and its molecular targets in a generalized neuron. TCAP may be liberated by either autoproteolytic cleavage from the C-terminus of the teneurin or by translation of a short mRNA from the free ribosomes. Soluble TCAP binds to the hormone-binding domain (HBD) of latrophilin-1 or-3 leading to combination with the dystroglycan complex where it stimulates a MEK-ERK phosphorylation response to regulate cytoskeleton organization and microtubule assembly. Further, TCAP binding to latrophilin may lead to Gα_s_ activation to stimulate cAMP and PKA targeting vesicular release. A major action of TCAP appears to act via Gα_q_ to elicit activation of the PLC-IP3 signal cascade to induce changes in cytosolic Ca^2+^ by acting on various Ca^2+^ channels to target mitochondrial function. This image was created using BioRender.

The three-dimensional structure of human (Li et al., 2018) and chicken (Jackson et al., 2018), teneurin-2 reveals that the extracellular region of teneurin possesses a unique bacteria “Tc” toxin-like organization. Bacterial Tc toxins are comprised of three proteins (A, B, C) where the “A” protein allows binding to target cells and the “B” and “C” proteins form a shell that protects the carboxy-terminal toxic component from the host (Busby et al., 2013). In typical fashion for such toxins, the teneurin extracellular domain contains prokaryotic-like YD repeats that form a spiralling “Barrel” domain made of up β-hairpins which act as a shell for a hydrophobic core (Jackson et al., 2018; Li et al., 2018). Unlike Tc toxins, where the toxin payload is protected in this shell, the C-terminal 120 amino acids of teneurin exit through a gap in the wall of the Barrel domain; this exposed “toxin-like” or “Tox-GHH” domain is therefore accessible for protein-protein interactions. Structurally, TCAP appears to be part of the toxin-like domain, occupying the distal part of an α-helical DNA-binding region at its N-terminus and forming a β-hairpin loop structure at its C-terminus, where unwinding of the helix could allow proteases to access and release soluble forms of TCAP (Jackson et al., 2018; Li et al., 2018; Figure 1).

## Cellular- and Organismal-Level Actions of Teneurin C-Terminal Associated Peptides in the Nervous System

Given evidence that the teneurin C-terminal region may be processed as a soluble peptide, synthetic forms of TCAP have been thoroughly probed for bioactivity at the cellular and organismal levels. Synthetic TCAP-1 has been shown to regulate cytoskeletal architecture in both immortalized and primary neuronal cell culture models. In N38 mouse immortalized hypothalamic cells, TCAP-1 treatment elicited increased process length but fewer processes per cell as well as increased β-actin and β-tubulin protein expression (Al Chawaf et al., 2007a). Similar increases in actin polymerization, expression of α-tubulin and β-tubulin, and process formation and length were observed in hippocampal E14 cells (Chand et al., 2012). TCAP-1 co-localizes with β-dystroglycan on the cell surface of E14 cells and may induce cytoskeletal rearrangement through the dystroglycan-associated mitogen-activated protein kinase-extracellular signal-regulated kinase 1/2 (MEK-ERK 1/2) pathway, leading to phosphorylation of stathmin at serine-25 and filamin A at serine-2152 to modulate microtubule formation and actin polymerization, respectively (Chand et al., 2012; Figure 1). *In vivo*, TCAP-1 treatment increased dendritic spine density in the CA1 and CA3 regions of the rat hippocampus (Tan et al., 2011). Together, these results indicate that TCAP-1 regulates neuronal function in a manner that modulates contacts between neurons through rearrangement of cytoskeletal elements. TCAP-1 also possesses neuroprotective actions, as it reduced alkalosis-associated necrotic cell death in N38 cells by upregulating expression of Cu-Zn superoxide dismutase 1 (SOD1) and catalase enzymes (Trubiani et al., 2007).

Teneurin C-terminal associated peptides provides sufficient cellular energy for actin and tubulin rearrangement in neurons through actions on metabolism (Hogg et al., 2018). *In vivo* studies in rats showed that TCAP-1 increased uptake of ^18^F-deoxyglucose in the frontal cortex and subcortical regions of the brain, which coincided with a decrease in serum glucose levels. Increased glucose uptake was also observed *in vitro* in TCAP-1-treated N38 cells, along with increased translocation of GLUT3 (the primary glucose transporter in the brain) to the plasma membrane, likely through activation of the phosphoinositide-3 kinase-protein kinase B (AKT) pathway and/or the MEK-ERK 1/2 pathway. TCAP-1 also increased intracellular ATP concentrations and decreased intracellular pyruvate and lactate concentrations in N38 cells, indicating that glucose metabolism through aerobic pathways was also upregulated.

These cellular-level changes offer a possible mechanistic explanation for the ability of TCAP-1 to modulate stress- and anxiety-related behaviours in rodents. TCAP-1-treated rats with high baseline emotionality (i.e., stress-sensitive) experienced a decrease in their acoustic startle response, a measure of their innate anxiety, whereas rats with low baseline emotionality (i.e., stress-insensitive) showed an increase, suggesting that TCAP-1 has a normalizing effect on anxiety (Wang et al., 2005). In elevated plus-maze experiments, another behavioural test for anxiety, TCAP-1-treated rats spent less time in the open arms, whereas rats treated with TCAP-1 and the stress-inducing hormone, corticotropin-releasing factor (CRF), showed a decrease in stretch-attend posture compared to those treated with CRF alone (Al Chawaf et al., 2007b; Tan et al., 2011) indicating again that TCAP treatment regulated anxiety in rodents. Similarly, TCAP-1 treatment significantly reduced CRF-induced expression of *c-Fos* in rat brain regions associated with anxiety (Tan et al., 2009). These results indicate that TCAP-1 may attenuate stress through functional antagonism of CRF actions.

## Involvement of Teneurin C-Terminal Associated Peptides in Intercellular Adhesion via the Teneurin-Latrophilin *Trans*-Synaptic Complex

Given its homology to CRF, TCAP was initially hypothesized to act as an antagonist on one or both of the CRF receptors (CRF-Rs) or another member of the Secretin family of G-protein coupled receptors (GPCRs) (see Qian et al., 2004; Wang et al., 2005). However, no such interactions have been reported to date. Although TCAP-1 co-localizes with β-dystroglycan, there is no direct evidence of a ligand-receptor interaction between the two molecules, indicating their co-localization may be part of a larger complex (Chand et al., 2012). More recently, the latrophilin (LPHN) family of Adhesion GPCRs, which are known *trans*-synaptic binding partners with the teneurins, have been identified as putative receptors for the TCAPs based on molecular evidence of their interaction. We therefore posit that soluble TCAP’s ability to modulate neuronal connectivity via cytoskeletal modulation occurs at least in part through latrophilin-associated G-protein signalling.

The latrophilins are members of the Adhesion family of GPCRs – a relatively unexplored class of GPCRs with unusually large extracellular domains that possess adhesive properties (review: Bjarnadóttir et al., 2007). Latrophilins were initially discovered as proteins with high affinity for α-latrotoxin, which is the main vertebrate-affecting neurotoxin in black widow spider (*Latrodectus tredecimguttatus*) venom (Frontali et al., 1976). The structure of latrophilin is typical of Adhesion GPCRs: they are type I-oriented with 7 looping transmembrane regions, a long N-terminal extracellular domain, and a shorter C-terminal cytoplasmic tail. In vertebrates, the extracellular region possesses several domains functional in cell adhesion, including a lectin-like domain (LEC) and an olfactomedin-like domain (OLF), which are involved in binding ligands such as the neurexins and fibronectin-like domain-containing leucine-rich transmembrane proteins (FLRTs; reviews: Silva and Ushkaryov, 2010; Meza-Aguilar and Boucard, 2014). Proximal to the cell membrane, the latrophilins possess a hormone-binding domain (HBD) which shares homology with CRF receptors and other Secretin family GPCRs, and is among several domains required for binding of α-latrotoxin (Holz and Habener, 1998; Krasnoperov et al., 1997, 1999; Ushkaryov et al., 2008). Intracellularly, the latrophilins interact with a variety of heterotrimeric G-proteins. Using α-latrotoxin affinity chromatography, LPHN-1 co-purified with Gα_o_ and Gα_q_ subunits *in vitro* (Rahman et al., 1999). Stimulation of LPHN-1-over-expressing cells with α-latrotoxin led to increased cAMP and inositol 1,4,5-triphosphate (IP_3_) production, where treatment with a phospholipase C (PLC) inhibitor eliminated α-latrotoxin-induced vesicular exocytosis (Lelianova et al., 1997; Davletov et al., 1998). Similarly, simian COS-7 cells overexpressing mouse LPHN-2 and certain splice variants of LPHN-3 experienced increases in IP_1_, the terminal metabolite of IP_3_, when stimulated with its self-derived “*Stachel*” peptide (Röthe et al., 2019). This indicates a functional role for the G_q/11_-protein signalling system, where intracellular calcium release from the endoplasmic reticulum evokes vesicular exocytosis characteristic of α-latrotoxin bioactivity (Lelianova et al., 1997). The G_i/o_, G_s_ and G_12/13_ families may also be involved in some contexts, as functional associations have been shown between human LPHN-1 and G_i_ or G_12/13_, between *C. elegans* LPHN-1 and Gα_s_, and between a pancreas-specific splice variant of LPHN-3 and G_i_ (Müller et al., 2015; Nazarko et al., 2018; Röthe et al., 2019).

The first evidence for the existence of a teneurin-latrophilin ligand-receptor interaction was established by Silva et al. (2011), where teneurin-2 expressed on post-synaptic dendritic branches bound to LPHN-1 expressed on pre-synaptic nerve terminals to form a *trans*-synaptic complex. Similar *trans*-cellular interactions were observed between teneurins-2 and 4 and all three latrophilins (Boucard et al., 2014) and between teneurin-1 and LPHN-3 (O’Sullivan et al., 2014). The LEC domain alone is sufficient for latrophilins to bind teneurins, but the interaction is strengthened by the OLF domain, and an alternative splice site between the LEC and OLF domains directly regulates latrophilin’s ability to bind to teneurins but not other ligands (O’Sullivan et al., 2012, 2014; Boucard et al., 2014; Meza-Aguilar and Boucard, 2014). There is some disagreement over which domains of the teneurins are involved in latrophilin binding. The initial study by Silva et al. (2011) found that Nb2a neuroblastoma cells over-expressing a C-terminal splice variant of teneurin-2 (which contains TCAP-2), named “Lasso,” heterophilically co-localized with cells over-expressing LPHN-1. In agreement with this, a truncated version of teneurin-2 which does not contain the C-terminal toxin-like domain was unable to bind to the LPHN-1 or 3 LEC domains (Li et al., 2018). More recent modelling of the trimeric teneurin-latrophilin-FLRT *trans*-synaptic complex by X-ray crystallography (del Toro et al., 2020) and cryo-EM (Li et al., 2020) suggest that the LPHN-2 and 3 LEC domains bind to the face of the teneurin-2 Barrel domain which is opposite to the TCAP-containing toxin-like region, such that the LEC and toxin-like regions are arranged in parallel but are separated by the Barrel domain. In the model by Li et al. (2018), the teneurin-2 toxin-like region was located approximately 6 nm away from the latrophilin-2 LEC binding surface. If the toxin-like region is in fact unnecessary for direct binding to latrophilin while associated with the full-length teneurin, perhaps cleavage at one of the accessible prohormone convertase sites allows for subsequent binding of a soluble C-terminal fragment to another domain within the latrophilin extracellular region. The teneurin-latrophilin *trans*-synaptic pairing via the YD-Barrel/LEC interaction would therefore ensure that any cleaved C-terminal fragment is in close proximity for diffusion to its putative receptive domain within the latrophilin extracellular region, and would explain evidence of downstream G-protein signalling upon treatment of cells over-expressing latrophilin with either Lasso or TCAPs (Silva et al., 2011; Vysokov et al., 2018; Husić et al., 2019). In agreement with this, Lasso triggers an increase in cytosolic calcium in cells over-expressing LPHN-1 and in pre-synaptic nerve terminals of hippocampal cells, which increased the rate of neurotransmitter exocytosis in a manner similar to α-latrotoxin (Silva et al., 2011; Vysokov et al., 2018).

Taken together these data suggest that, like α-latrotoxin, Lasso signals through G_q/11_, and this agonist activity has been implicated in axonal pathfinding in the developing hippocampus (review: Ushkaryov et al., 2019). Similarly, latrophilins may act as receptors for free soluble TCAPs. Human embryonic kidney 293 (HEK 293) cells over-expressing LPHN-1 demonstrate significantly higher co-localization with fluorescently tagged recombinant TCAP-1 compared to wild-type cells, as well as greater cytoskeletal remodelling in response to TCAP-1 (Husić et al., 2019). Given latrophilin’s homology to the CRF-Rs, we posit that the latrophilin HBD is responsible for TCAP binding. Cells transfected with a mutated LPHN-1 construct that does not contain the HBD showed minimal co-localization with TCAP-1, and TCAP-1 co-immunoprecipitated with isolated HBD constructs *in vitro* (Husić et al., 2019). Like α-latrotoxin and Lasso, TCAP-1 may utilize the G_q/11_ signalling pathway. However, since TCAP-1 has also been shown to modulate intracellular cAMP, so G_i/o_ and G_s_ could also be involved (Qian et al., 2004; Wang et al., 2005). Given that there are 4 isoforms of TCAP in vertebrates and 3 of LPHN, and numerous splice variants and some interspecies variation within these genes, there are many possible combinations of TCAP-latrophilin interactions which could account for TCAP’s diverse actions.

## Evolution of Cell Adhesion via the Teneurin/Teneurin C-Terminal Associated Peptides-Latrophilin Complex

The teneurins and TCAPs are evolutionarily ancient and conserved across metazoans. The teneurin genes likely evolved through the horizontal gene transfer (HGT) of a polymorphic proteinaceous toxin (PPT) gene from an aquatic prokaryote to a choanoflagellate, which is a unicellular ancestor to metazoans (Tucker et al., 2012; Zhang et al., 2012). Functionally, the ancestral teneurin gene may have assisted choanoflagellates in food acquisition, given the adhesive properties of the teneurin extracellular domains (Tucker et al., 2012; Tucker, 2018). Moreover, the presence of 4 teneurin genes in vertebrates supports the “2R” hypothesis, which postulates that 2 genome duplication events occurred during the course of vertebrate evolution (Ohno, 1970; see review: Lovejoy et al., 2006). Like much of the C-terminal region of teneurin, TCAP appears to have a prokaryotic origin and possibly represents an inactive form of the toxin payload from a PPT (Chand et al., 2013). TCAP also shares homology with (but evolutionarily predates) the CRFs, calcitonins and Secretin family peptides, and has approximately 20% sequence similarity with α-latrotoxin (Lovejoy et al., 2006; Michalec et al., 2020). Thus, the ancestral toxin-like gene acquired by HGT could have given rise to all of these peptides. We postulate that in early metazoans, CRF-like and TCAP-like genes worked antagonistically as a rudimentary stress and metabolism control system. This is supported by findings in the chordate species, vase tunicate (*Ciona intestinalis*), where CRF/diuretic hormone-like peptide and TCAP oppositely modulated feeding behaviours (D’Aquila et al., 2017).

The latrophilins are similarly omnipresent in the metazoans, indicating that like the teneurins, they were acquired early in evolutionary history. Upon discovery, latrophilins were initially classified as Secretin family GPCRs due to their possession of an HBD, but they have since been re-classified as members of the Adhesion family (Lelianova et al., 1997). Evolutionarily, the Adhesion family of GPCRs are among the oldest GPCRs, even appearing in unicellular fungi, though the extracellular adhesive domains associated with latrophilins such as the LEC, OLF and HBD evolved later (Krishnan et al., 2012; Schöneberg and Prömel, 2019). The latrophilin OLF domain is found only in the vertebrate lineage, and while some modern invertebrates possess LEC and HBD, they are poorly conserved, so it remains uncertain if early metazoan latrophilins would be capable of *trans*-cellular interactions with teneurins on their own, and instead may rely on interactions through a complex with other adhesion proteins (Woelfle et al., 2015; Schöneberg and Prömel, 2019). Interestingly, the Adhesion GPCRs precede the Secretin GPCRs, and comparably, TCAP is a predecessor to the peptide family that binds to Secretin GPCRs (Lovejoy et al., 2006; Nordström et al., 2009). Thus, the ancestral teneurin/TCAP and latrophilins genes could have given rise to many of the peptide signalling systems present in modern organisms.

Given the evolutionarily ancient history of the teneurins, TCAPs, and latrophilins, there is a high probability that this protein system is functional in a number of tissues across the metazoans. Since early organisms possessing these genes did not have complex nervous systems, there were likely some other evolutionary advantages independent from the described modern role in neuronal cell adhesion. The adhesive properties of both teneurins and latrophilins could assist in development of non-neuronal tissues that require precise organization and structure. Furthermore, the conserved energy regulatory actions of TCAP would be particularly advantageous in tissues that have high energy demands, such as those involved in locomotion.

## Conclusion

The teneurin-latrophilin *trans*-synaptic pair represents an evolutionarily ancient mechanism for cell adhesion that has been co-opted for neuronal wiring among the vertebrates. At the molecular level, the most recent modelling suggest that the TCAP region does not directly bind to latrophilin while still affiliated with the full-length teneurin protein, but soluble TCAP either cleaved from teneurin or separately transcribed may have a functional role in latrophilin signalling through interaction with the hormone binding domain (Husić et al., 2019; del Toro et al., 2020; Li et al., 2020). There is a clear functionality for soluble TCAP in modulating neuronal contacting in culture, which offers a likely cellular-level explanation for its ability to modulates stress- and anxiety-related behaviours *in vivo*.

## Author Contributions

TD wrote the drafts of the manuscript. DL oversaw the completion of the final version. Both authors contributed to the article and approved the submitted version.

## Conflict of Interest

The authors declare that the research was conducted in the absence of any commercial or financial relationships that could be construed as a potential conflict of interest.

## Publisher’s Note

All claims expressed in this article are solely those of the authors and do not necessarily represent those of their affiliated organizations, or those of the publisher, the editors and the reviewers. Any product that may be evaluated in this article, or claim that may be made by its manufacturer, is not guaranteed or endorsed by the publisher.
